# Effect of *Thunbergia laurifolia* Herbal Tea on Glucose Homeostasis in Healthy Volunteers: A Single-Arm Phase I Study

**DOI:** 10.1155/2020/3212546

**Published:** 2020-07-23

**Authors:** Lukana Preechasuk, Pravit Akarasereenont, Ranida Boonrak, Onusa Thamsermsang, Busadee Pratumvinit, Nuntakorn Thongtang

**Affiliations:** ^1^Siriraj Diabetes Center of Excellence, Faculty of Medicine Siriraj Hospital, Mahidol University, Bangkok 10700, Thailand; ^2^Center of Applied Thai Traditional Medicine, Faculty of Medicine Siriraj Hospital, Mahidol University, Bangkok 10700, Thailand; ^3^Department of Pharmacology, Faculty of Medicine Siriraj Hospital, Mahidol University, Bangkok 10700, Thailand; ^4^Department of Clinical Pathology, Faculty of Medicine Siriraj Hospital, Mahidol University, Bangkok 10700, Thailand; ^5^Division of Endocrinology and Metabolism, Department of Medicine, Faculty of Medicine Siriraj Hospital, Mahidol University, Bangkok 10700, Thailand

## Abstract

**Background:**

*Thunbergia laurifolia* (TL) is a commonly used herbal medicine in Thailand and in other Asian countries. TL has been approved as a Thai traditional medicine for detoxifying poisons, and the list of possible adverse effects includes hypoglycemia. TL showed hypoglycemic effect in animals possibly due to antioxidant effect and beta-cell preservation. However, the safety of TL herbal tea and its effects on glucose homeostasis have never been investigated in humans.

**Methods:**

Twenty healthy volunteers (10 men and 10 women) drank TL herbal tea 3 times/day for 2 weeks. Ten subjects took TL herbal tea 9 grams daily. After the safety of TL herbal tea was established, 10 more subjects took TL 12 grams daily. Clinical and biochemical tests were assessed at baseline and at 2 weeks.

**Results:**

Mean age was 34.9 ± 10.2 years, and mean body mass index was 27.5 ± 5.8 kg/m^2^. Baseline and posttreatment plasma concentrations were as follows: fasting plasma glucose (89 ± 6 vs. 89 ± 7 mg/dL), fructosamine (213 ± 32 vs. 212 ± 33 *μ*mol/L), fasting insulin (8.8 [IQR: 5.9–18.4] vs. 10.4 [IQR: 7.4–15.2] *μ*U/mL), HOMA-B (101.6 [IQR: 82.3–189.8] vs. 120.4 [IQR: 93.2–153.2]), and HOMA-IR (1.1 [IQR: 0.8–2.3] vs. 1.4 [IQR: 0.9–2.0]), all respectively. There were no significant changes in these parameters, including body weight, blood pressure, lipid profile, and C-reactive protein. No serious adverse events were observed during the study period.

**Conclusions:**

TL herbal tea at doses of 9 and 12 grams daily had good tolerability without any significant adverse effects on fasting plasma glucose level or other glucose homeostasis parameters measured.

## 1. Introduction

*Thunbergia laurifolia* (TL) is a commonly used herbal medicine in Thailand and Indonesia. It is commonly referred to as blue trumpet vine or “Rang Chuet” in Thai language. TL is an ornamental plant that belongs to the Acanthaceae family [1, 2]. There are phytochemicals in plants that are biologically active, naturally occurring chemical compounds that provide health benefits for humans as medicinal ingredients and nutrients [3]. Compounds that are isolated from the leaves of TL include phenolic acids (chlorogenic acid, caffeic acid, gallic acid, and protocatechuic acid), flavonoids (apigenin and grandifloric acid), and glycosides (iridoid glycoside and glucopyranosides) [4]. Active substances in TL were found to have antioxidant activities. Animal studies showed that TL can alleviate toxicity from lead [5] and cadmium [6]. In addition, TL significantly decreased toxicity in parathion- and paraquat-intoxicated rats [7, 8]. Previous animal studies also reported that TL can reduce toxicity from alcohol in the liver [9, 10]. As a result of these reported health benefits, TL has been approved as a modified Thai traditional drug on the Thai National List of Essential Medicines (NLEM) for detoxification of poisons and for its antipyretic property. TL is commonly used in Thai traditional medicine clinics to detoxify pesticides and alcohol, and it is delivered in the form of an herbal tea that is consumed orally. Among the 74 Thai traditional drugs on the NLEM, TL is the only drug that is labeled with hypoglycemia being a possible side effect. More than 400 plants and compounds demonstrate antidiabetic activities via diverse mechanisms, such as reduced insulin resistance, improved beta-cell function, incretin-related pathway, and delaying glucose absorption [11]. *In vitro* [12] and animal studies [13, 14] demonstrated that TL has antioxidant effect and antidiabetic effect and possibly preserves beta-cell function. However, no previous studies investigated the hypoglycemic effect of TL in humans. Accordingly, the aim of this study was to investigate the effect of TL on glucose homeostasis and to assess the safety of TL use in humans.

## 2. Materials and Methods

### 2.1. Subjects

Twenty healthy volunteers (10 men and 10 women) aged 20 to 60 years were recruited during the October 2018 to February 2019 study period. This prospective pilot study was conducted at the Faculty of Medicine Siriraj Hospital, Mahidol University. Siriraj Hospital is a 2,300-bed university-based national tertiary referral center that is located in Bangkok, Thailand. The inclusion criteria were volunteers who had no underlying disease, did not take any medications, and had no allergic history to TL use. The exclusion criteria were elevation of transaminase enzyme higher than 2-fold of the upper normal limit or plasma creatinine higher than 1.5 mg/dL.

### 2.2. Study Drug

#### 2.2.1. Preparation of TL Herbal Tea

TL herbal tea was manufactured under good manufacturing practice guidelines by the Herbal Medicine and Products Manufacturing Unit, Center of Applied Thai Traditional Medicine, Faculty of Medicine Siriraj Hospital, Mahidol University (Bangkok, Thailand). The manufacturing unit was approved for the Pharmaceutical Inspection Cooperation Scheme Good Manufacturing Practice from the Thailand Food and Drug Administration. TL was sourced from contract suppliers and/or contract farmers, and its authenticity was confirmed by experts from Center of Applied Thai Traditional Medicine. The raw materials were washed with deionized water and dried in a hot air oven in accordance with TL herbal tea. The TL leaves were then mashed, packed in a tea bag (1 gram of TL per bag), and stored at 25°C with 60% relative humidity.

#### 2.2.2. Chemical Screening of TL Using Ultra-Performance Liquid Chromatography Analysis

**Figure 1:**
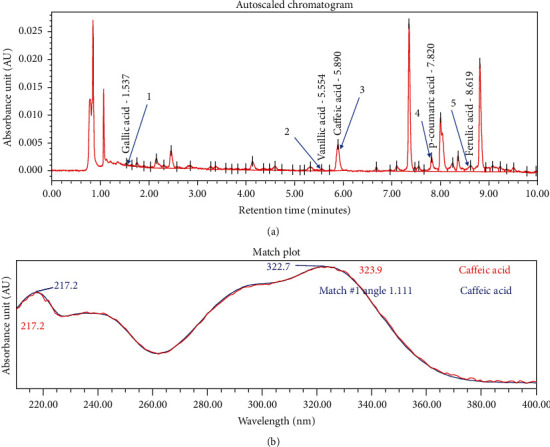
Chromatogram of *Thunbergia laurifolia* using ultra-performance liquid chromatography (UPLC) analysis. (a) UPLC fingerprint of the extract of *Thunbergia laurifolia* under PDA 280 nm. Peak 1 (gallic acid) retention time (RT): 1.537 min; peak 2 (vanillic acid) RT: 5.554 min; peak 3 (caffeic acid) RT: 5.890 min; peak 4 (p-coumaric acid) RT: 7.820 min; and peak 5 (ferulic acid) RT: 8.619 min. (b) spectrum of *Thunbergia laurifolia* extract that matched with caffeic acid standard.

Ultra-performance liquid chromatography (UPLC) analysis was performed using an ACQUITY UPLC BEH C18 Column (100 mm × 2.1 mm I.D, particle size of 1.7 *μ*m; Waters Corporation, Milford, MA, USA). Five phenolic standards comprising of gallic acid, vanillic acid, caffeic acid, p-coumaric acid, and ferulic acid were used as reference makers in TL tea. Photodiode array (PDA) detection was performed on a Waters ACQUITY UPLC® System (Waters Corporation) equipped with a binary solvent delivery system, an online degasser, an autosampler, and a thermostatically controlled column system. TL methanolic extract separated in reverse phase of 0.1% O-phosphoric acid : acetonitrile (95 : 5) showed the fingerprint demonstrated in Figure 1(a). The chromatogram only matched with caffeic acid spectrum and was confirmed with the authentic standard at 280 nm (Figure 1(b)).

### 2.3. Study Design

The recommended dose of TL powder formula to detoxify poison that is listed in the NLEM is 2 to 3 grams three times per day. However, the dosage of TL to assess its hypoglycemic effect has never been investigated in humans. The dosage of TL that was found to decrease blood glucose level in alloxan-induced diabetic rats was 60 mg/ml/day of aqueous extract from the leaves (rat body weight was 100–150 grams) [13]. The TL dose for human (∼70 kg) that was calculated from the rat study using allometric scaling calculator [15] should be at least 6–8 grams per day. However, dosages of 9 and 12 grams daily were tested in the present study based on the recommended dose from the NLEM. This study was performed in 2 phases. The volunteers from both phases were recruited independently. In the first phase, 10 subjects (5 men and 5 women) received TL herbal tea 9 grams daily for 2 weeks. After the safety of TL herbal tea was established, 10 more subjects (5 men and 5 women) took TL herbal tea 12 grams daily for 2 weeks. Subjects drank TL herbal tea 3 times daily before each meal. TL herbal tea was brewed for 5 minutes using 200 ml of hot water. After 12-hour overnight fast, subjects came to the Clinical Research Unit of the Division of Endocrinology and Metabolism, Department of Medicine, Faculty of Medicine Siriraj Hospital, Mahidol University, on day 1 and day 14 of each phase.

### 2.4. Outcome Variables

**Figure 2:**
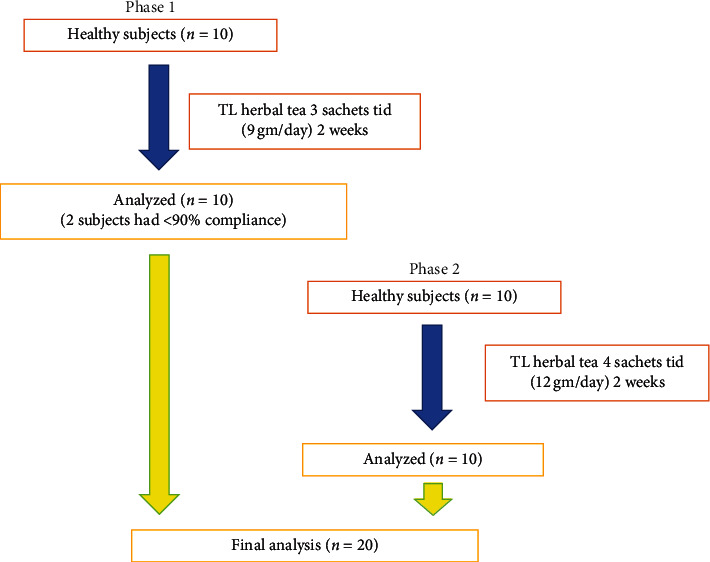
Study flow chart.

History taking and physical examination, including body weight, blood pressure, and biochemical tests, were assessed at baseline and at 2 weeks for each phase. Adverse events were also assessed at 2 weeks (Figure 2). Fasting plasma glucose (FPG), cholesterol, triglycerides, high-density lipoprotein cholesterol (HDL-C), creatinine, total bilirubin, aspartate transaminase, alanine transaminase, high-sensitivity C-reactive protein (hs-CRP), fasting insulin, and cortisol were measured on a cobas® 8000 modular analyzer (Roche Diagnostics, Basel, Switzerland). Plasma low-density lipoprotein cholesterol (LDL-C) levels were calculated using Friedewald formula. Complete blood count was analyzed using an XN-3000 automated hematology analyzer (Sysmex Corporation, Kobe, Japan), and urinalysis was performed using LabUMat 2 and UriSed 2 automated urine chemistry analyzers (77 Elektronika, Budapest, Hungary). Fructosamine was analyzed using Architect 16000 (Abbott Laboratories, Chicago, IL, USA). Fructosamine was used in this study because it can be used to test changes in plasma glucose within a two-week period.

Steady-state beta-cell function (B), insulin sensitivity (S), and insulin resistance (IR) were calculated using Homeostasis Model Assessment (HOMA) 2 calculator software of The Diabetes Trials Unit, The Oxford Center for Diabetes, Endocrinology and Metabolism (https://www.dtu.ox.ac.uk/homacalculator/). The protocol for this study was approved by the Siriraj Institutional Review Board (COA no. Si 755/2017), and all subjects provided written informed consent. The trial registration number is TCTR 20190809004.

### 2.5. Statistical Analysis

All statistical analyses were performed using IBM SPSS Statistics for Windows, version 20.0 (IBM Corp., Armonk, NY, USA). Continuous data are reported as mean ± standard deviation (SD) or median and interquartile range (IQR), and categorical data are presented as number and percentage. Continuous data were analyzed using paired samples *t*-test or Wilcoxon signed-rank test, and categorical data were compared using chi-square test or Fisher's exact test, as appropriate. A *p* value of <0.05 was considered statistically significant.

For the participant pattern analysis, eleven clinically significant parameters for glucose homeostasis, plasma lipids, and inflammatory markers were used. These parameters include FPG, fasting insulin, fructosamine, HOMA-B, HOMA-S, HOMA-IR, total cholesterol, triglyceride, HDL, LDL, and hs-CRP before and after TL herbal tea administration. Briefly, all raw data were converted to a comma-separated values (CSV) file in Microsoft Excel program for Windows and then imported into ClustVis, which is a web tool for visualizing clustering of multivariate data (https://biit.cs.ut.ee/clustvis/) [15]. The results of that analysis are presented as a heatmap with correlational analysis and a principal component analysis (PCA) plot.

## 3. Results

### 3.1. Demographic Data and Biochemical Parameters of the Study Patients

The mean age of volunteers was 34.9 ± 10.2 years, and the mean body mass index was 27.5 ± 5.8 kg/m^2^. Study volunteers had a normal FPG level at baseline, with a mean FPG level of 89 ± 6 mg/dl. At baseline, all participants were normotensive and relatively insulin sensitive with a median HOMA-IR of 1.1 (less than 2.0). After 2 weeks of TL herbal tea consumption at 9 grams (10 subjects) and 12 grams (the other 10 subjects) daily, there were no significant changes in body weight, blood pressure, glucose homeostasis, lipid profile, or hs-CRP levels, as shown in Table 1. No significant changes from baseline in these parameters were observed when the results of both phases were combined (*n* = 20).

Unused teabags were counted to assess treatment adherence. Two male volunteers in the 9 gm/day phase had poor adherence (less than 90% of TL tea consumption). One participant reported that he forgot to take the tea before some meals, and the other forgot to bring the teabags with him while travelling to another province. The results of these metabolic profiles were unchanged when we calculated excluding 2 subjects who had poor compliance (*n* = 18).

No serious adverse events were observed or reported during the study period including the 2 participants who had poor compliance. There were no significant changes from baseline in hemoglobin, platelet count, kidney function, liver function test, or plasma cortisol level after taking TL herbal tea for 2 weeks, as shown in Table 2. White blood cell count was slightly elevated after treatment in the TL 9 grams daily group but this effect was not seen in the 12 grams daily group.

### 3.2. The Effects of TL Herbal Tea on the Metabolic Patterns in Blood

**Figure 3:**
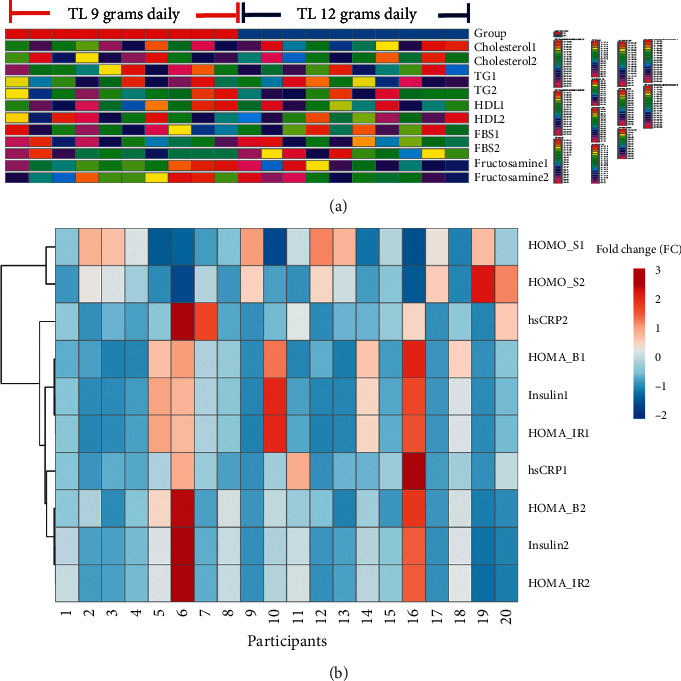
Clustergram of all participants before and after TL administration at different doses. Parameters with number 1 indicate baseline values and number 2 indicate values at 2 weeks after TL administration. (a) Raw data of each participant. Red color indicates greater numbers while pink color indicates lesser numbers. (b) Fold changes of each parameter between −2 and 3. Red color indicates greater values while blue color indicates lesser values.

The clustergram shown in Figure 3, which is a hierarchical cluster analysis heatmap, shows differences in metabolic patterns between participants who received TL herbal tea 9 grams and 12 grams daily (Figure 3).  Figure 3(a) from cholesterol to fructosamine parameters represents raw data of each participant. Red color indicates greater numbers while pink color indicates lesser numbers.  Figure 3(b) represents fold changes of each parameter. Red color indicates greater values while blue color indicates lesser values range from +3 to −2 fold changes. Some slight changes in metabolic parameters before and after TL herbal tea administration were observed within group. For example, the color shades of HOMA sensitivity (HOMA-S) 2 changed to higher values than the color shades of HOMA-S1 which indicates improvement in insulin sensitivity after treatment in participants numbers 7 and 10 treated with TL 9 grams daily and participants numbers 14, 17, 19, and 20 treated with TL 12 grams daily. The color shades of HOMA-beta (HOMA-B) 2 changed to higher values than the color shades of HOMA-B1 which indicated improvement in beta-cell function after TL treatment in participants numbers 2, 4, 6, and 8 with TL 9 grams daily and participant number 11 with TL 12 grams daily. For correlational analysis, several metabolic factors also relatively contributed to one another such as fasting insulin, HOMA-IR, HOMA-B, and high-sensitivity CRP.

### 3.3. Discrimination between Different Doses of TL Herbal Tea in Healthy Volunteers

**Figure 4:**
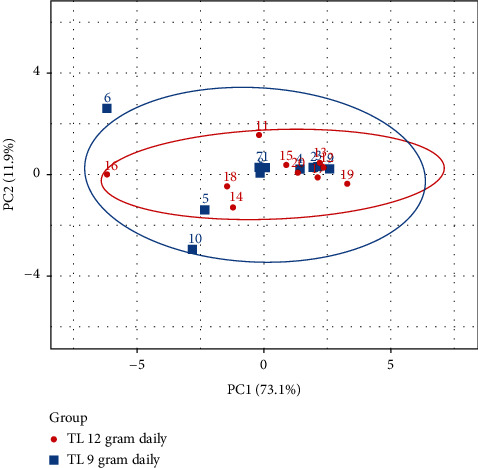
Discrimination analysis of participants who received TL herbal tea 9 grams/daily (blue dot) and 12 grams/daily (red dot). Each dot represents multivariate analysis of an individual subject using ClustVis program. The *x*-axis and *y*-axis represent principle component 1 (PC1) and principle component 2 (PC2), respectively.

Clinical and inflammatory parameters from blood profiling of participants (plasma FPG, fasting insulin, fructosamine, HOMA-B, HOMA-S, HOMA-IR, total cholesterol, triglyceride, HDL, LDL, and hs-CRP) were entered into multivariate analysis. Principal component analysis revealed significant differences in parameters analyzed between healthy volunteers who received TL herbal tea 9 grams daily and those who received 12 grams daily (*p* < 0.05). Principle components (PC1 and PC2) accounted for 73.1% and 11.9% of the total variables, respectively, indicating that there was a discrimination between 9 and 12 grams daily doses of TL herbal tea on the effects of these parameters analyzed (Figure 4). When we performed analysis including only 18 subjects who had good compliance, the discrimination between different doses was more pronounced. Principle components (PC1 and PC2) accounted for 74.0% and 13.1% of the total variables, respectively (*n* = 18).

## 4. Discussion

We found that TL herbal drinking tea, which is labeled as having risk for hypoglycemia, had no adverse effects on fasting plasma glucose, HOMA-beta, or HOMA-IR in healthy volunteers at dosages of 9 and 12 grams daily for 2 weeks.

In addition to the antioxidant and detoxification properties [5–10], hypoglycemic effects of TL were reported in animal studies. Aritajat et al. [13] reported that the blood glucose level of alloxan-induced diabetic rats decreased significantly after 15 days of TL extract treatment (60 mg/ml/day); however, there was a nonsignificant trend of decreased blood glucose level in normoglycemic rats. The islets of Langerhans of diabetic rats treated with TL extract were larger and had more *β*-cells compared to the untreated group. As a result of the severe destruction of *β*-cells in alloxan-induced diabetic rats, the glucose lowering effect in that study may have been due to insulin-like substances in TL leaf, or due to substances that induce the recovery of *β*-cells to secrete insulin. Pitoolpong et al. [14] studied the effect of TL extract in hyperglycemic cats whose high glycemic level was induced by a high carbohydrate diet. After 4 weeks of TL extract treatment, blood glucose level and area under the curve of glucose from intravenous glucose tolerance test of hyperglycemic cats decreased significantly. Since there were no data regarding *β*-cell function, the mechanisms underlying the observed hypoglycemic effect in that study remain unclear.

Although we did not find any hypoglycemic effects of TL drinking tea in healthy volunteers, the hypoglycemic effects of TL in patients with diabetes cannot be excluded because hypoglycemic effects were previously demonstrated in hyperglycemic animal models, but not in normoglycemic models. To the best of our knowledge, this is the first study to investigate the effect of TL herbal tea on glucose homeostasis parameters in human. Moreover, we sought to assess safety profile of TL herbal tea. There were no serious adverse events observed or reported by any study volunteer after taking TL herbal drinking tea at dosages of 9 or 12 grams daily. There were also no significant changes in liver or renal function test. Moreover, complete blood count and basal plasma cortisol levels were unchanged after 2 weeks of TL herbal tea consumption.

The phytochemical substances found in TL were phenolics (caffeic acid, gallic acid, and protocatechuic), flavonoids (apigenin), glycosides, and alkaloids [1]. Caffeic acid, the phytochemical substance found in our study, has been shown to have antihyperglycemic effect in studies of both streptozotocin-induced diabetic rat (STZ-rat) and insulin resistant rat (IR-rat). Hsu et al. found that single dose intravenous injection of caffeic acid reduced plasma glucose and increased glucose uptake of adipocyte in STZ-rat and IR-rat [16]. Zahran et al. reported that 30-day administration of caffeic acid reduced blood glucose, increased insulin and antioxidants levels, and restored pancreatic histology in streptozotocin-diabetic rats compared to control diabetic rats [17]. Jung et al. studied the effect of caffeic acid supplementation for 5 weeks in IR-mice. They found that mice in caffeic acid group had lower plasma glucose, glycated hemoglobin, and glucagon level and had higher plasma insulin, leptin, and antioxidant level with more preserved islet architecture as compared with the control group. These effects could be from an attenuation of hepatic glucose output and an enhancement of adipocyte glucose uptake [18]. Apigenin, which is a subclass of flavonoids, also demonstrated antidiabetic effect in diabetic rats [19–21] with various proposed mechanisms, such as antioxidant effect [22], enhancing GLUT4 translocation [20], inhibiting DPP4 enzyme [23], and protective effect on pancreatic beta-cell destruction [20]. Furthermore, *in vitro* study demonstrated that TL had inhibitory activity against alpha amylase enzyme [24]. This inhibitory activity should delay carbohydrate degradation and absorption, which should result in lower blood glucose levels. Future study that investigates the effect of TL in diabetic patients is needed. Based on our principal component analysis result, which indicated significant differences in changes of glucose, lipids, and inflammatory markers analyzed between healthy volunteers who received TL herbal tea 9 grams daily and those who received TL 12 grams daily, the dosage of 12 grams daily should be selected for the future studies.

### 4.1. Limitations

First, our study had a small sample size because it was a pilot study and TL has never been previously studied in humans. Second, our follow-up duration from baseline was only two weeks. These two factors may have limited the ability of our study to identify statistically significant changes in the evaluated parameters from baseline to the end of the study. In addition, we investigated the effects of TL in healthy volunteers; thus, it is possible that TL may not have the same neutral effect on glucose homeostasis in people with diabetes. Animal studies found hypoglycemic effects only in rats with diabetes, but no effects were observed in rats without diabetes. Moreover, the dosage of TL that might have effects on glucose homeostasis in human is currently unknown; thus we used the dosage that was commonly used for detoxifying poison in our study. However, we suggested that future studies should investigate the effects of TL at a dose of 12 grams daily. Additional studies investigating mechanism of actions of TL on glucose and lipid metabolism in human should be done.

## 5. Conclusion

TL herbal tea at doses of 9 and 12 grams per day for 2 weeks did not increase the risk of hypoglycemia in healthy volunteers. TL had no effects on fasting plasma glucose levels, insulin secretion (HOMA-beta), or insulin resistance (HOMA-IR) in healthy volunteers.

## Figures and Tables

**Table 1:** Glucose homeostasis and metabolic profiles at baseline and at 2 weeks.

	All (*n* = 20)			TL 9 gm/day (*n* = 10)			TL 12 gm/day (*n* = 10)		
	Baseline	2 weeks	*p* value	Baseline	2 weeks	*p* value	Baseline	2 weeks	*p* value
Weight (kg)	72.1 ± 16.3	72.2 ± 16.2	0.71	73.0 ± 17.4	72.8 ± 17.1	0.60	71.3 ± 16.1	71.6 ± 16.3	0.17
BMI (kg/m^2^)	27.5 ± 5.8	27.6 ± 5.8	0.63	28.1 ± 6.0	28.0 ± 5.9	0.68	27.0 ± 6.0	27.1 ± 6.0	0.17
SBP (mmHg)	110 ± 14	108 ± 11	0.52	107 ± 13	107 ± 10	0.96	116 ± 14	110 ± 12	0.30
DBP (mmHg)	66 ± 10	67 ± 10	0.77	65 ± 8	68 ± 11	0.48	67 ± 12	66 ± 9	0.58
FPG (mg/dL)	89 ± 6	89 ± 7	0.80	90 ± 5	89 ± 6	0.36	88 ± 6	90 ± 8	0.24
Fructosamine (*μ*mol/L)	213 ± 32	212 ± 33	0.94	194 ± 29	194 ± 24	0.93	231 ± 25	231 ± 32	0.99
Fasting insulin (*μ*U/mL)	8.8 (5.9–18.4)	10.4 (7.4–15.2)	0.72	10.5 (5.9–22.9)	11.7 (7.9–15.3)	0.79	7.9 (5.8–17.0)	9.5 (6.5–15.6)	1.00
HOMA-B	101.6 (82.3–189.8)	120.4 (93.2–153.2)	0.77	113.4 (81.0–201.4)	130.2 (97.3–161.3)	0.45	96.2 (83.7–184.5)	104.1 (85.8–137.4)	0.51
HOMA-S	88.2 (43.6 130.3)	73.8 (50.7–107.0)	0.31	73.4 (34.9–130.4)	68.5 (51.7–97.8)	0.24	95.6 (47.1–132.3)	81.2 (49.4–120.3)	0.96
HOMA-IR	1.14 (0.77–2.31)	1.37 (0.94–1.98)	0.65	1.37 (0.77–2.87)	1.50 (1.03–1.94)	0.72	1.05 (0.76–2.14)	1.27 (0.84–2.03)	0.88
Cholesterol (mg/dL)	195 ± 28	200 ± 29	0.25	198 ± 24	201 ± 34	0.67	192 ± 32	199 ± 25	0.67
TG (mg/dL)	91 ± 28	97 ± 38	0.24	96 ± 32	104 ± 45	0.29	86 ± 25	91 ± 30	0.29
HDL-C (mg/dL)	52 ± 9	53 ± 11	0.35	49 ± 8	50 ± 11	0.42	54 ± 9	55 ± 10	0.42
Cal-LDL-C (mg/dL)	125 ± 28	128 ± 27	0.50	130 ± 26	130 ± 31	0.96	121 ± 31	126 ± 25	0.96
hs-CRP (mg/L)	1.49 (0.72–2.63)	1.31 (0.58–3.20)	0.77	1.43 (0.71–2.44)	1.26 (0.78–3.31)	0.26	1.73 (0.61–3.87)	1.58 (0.44–3.78)	0.14

Data presented as mean ± standard deviation or median and interquartile range. A *p* value <0.05 indicates statistical significance. Abbreviations: BMI, body mass index; SBP, systolic blood pressure; DBP, diastolic blood pressure; FPG, fasting plasma glucose; HOMA-B, homeostatic model assessment of *β*-cell function; HOMA-S, homeostatic model assessment of insulin sensitivity; HOMA-IR, homeostatic model assessment of insulin resistance; HDL, high-density lipoprotein cholesterol; cal-LDL-C, calculated low-density lipoprotein cholesterol; hs-CRP, high sensitivity C-reactive protein.

**Table 2:** Biochemical and hematological parameters at baseline and at 2 weeks.

	All (*n* = 20)			TL 9 gm/day (*n* = 10)			TL 12 gm/day (*n* = 10)		
	Baseline	2 weeks	*p* value	Baseline	2 weeks	*p* value	Baseline	2 weeks	*p* value
Cortisol (ug/dL)	8.0 ± 2.6	8.2 ± 3.1	0.84	7.6 ± 2.8	7.0 ± 2.3	0.42	8.5 ± 2.4	9.3 ± 3.5	0.43
eGFR (ml/min/m^2^)	114 ± 23	107 ± 15	0.23	107 ± 14	106 ± 15	0.57	120 ± 29	108 ± 16	0.27
AST (U/L)	18 (16–24)	20 (16–24)	0.87	19 (16–25)	20 (18–23)	0.59	18 (14–25)	28 (16–28)	0.86
ALT (U/L)	15 (10–31)	16 (11–24)	0.62	14 (9–37)	16 (13–25)	0.44	16 (11–23)	15 (11–23)	0.86
TB (mg/dL)	0.5 (0.4–0.7)	0.5 (0.3–0.8)	0.47	0.6 (0.3–0.7)	0.6 (0.3–0.8)	0.58	0.5 (0.4–0.7)	0.5 (0.3–0.5)	0.06
Hematocrit (%)	39.3 ± 5.4	39.7 ± 4.5	0.46	39.2 ± 6.1	39.2 ± 4.4	1.00	39.4 ± 4.9	40.2 ± 4.8	0.07
WBC (cells/mm^3^)	5746 ± 1314	6212 ± 921	0.07	5365 ± 971	6130 ± 958	0.04	6126 ± 1542	6293 ± 926	0.66
Platelets (×10^3^/*μ*L)	274 ± 64	284 ± 69	0.35	269 ± 67	277 ± 79	0.49	280 ± 65	290 ± 62	0.54

Data are presented as mean ± standard deviation or median and interquartile range. A *p* value <0.05 indicates statistical significance. Abbreviations: eGFR, estimated glomerular filtration rate; AST, aspartate transaminase; ALT, alanine transaminase; TB, total bilirubin; WBC, white blood cell count.

## Data Availability

The dataset used and/or analyzed during the current study is available from the corresponding author upon reasonable request.

## Abbreviations

AST: Aspartate aminotransferase
ALT: Alanine aminotransferase
BUN: Blood urea nitrogen
CBC: Complete blood count
Cr: Creatinine
FPG: Fasting plasma glucose
hs-CRP: High-sensitivity C-reactive protein
TB: Total bilirubin
TL: *Thunbergia laurifolia*
UA: Urinalysis.
